# Patient or treatment centre? Where are efforts invested to improve cancer patients' psychosocial outcomes?

**DOI:** 10.1111/j.1365-2354.2010.01211.x

**Published:** 2011-03

**Authors:** ML Carey, T Clinton-McHarg, RW Sanson-Fisher, S Campbell, HE Douglas

**Affiliations:** 1 The Priority Research Centre for Health Behaviour, Faculty of Health, The University of NewcastleNSW, Australia; 2 Centre for Behavioral Research and Program Evaluation, University of WaterlooON, Canada

**Keywords:** cancer, psychosocial, systems, psychological, quality of life

## Abstract

The psychosocial outcomes of cancer patients may be influenced by individual-level, social and treatment centre predictors. This paper aimed to examine the extent to which individual, social and treatment centre variables have been examined as predictors or targets of intervention for psychosocial outcomes of cancer patients. Medline was searched to find studies in which the psychological outcomes of cancer patient were primary variables. Papers published in English between 1999 and 2009 that reported primary data relevant to psychosocial outcomes for cancer patients were included, with 20% randomly selected for further coding. Descriptive studies were coded for inclusion of individual, social or treatment centre variables. Intervention studies were coded to determine if the unit of intervention was the individual patient, social unit or treatment centre. After random sampling, 412 publications meeting the inclusion criteria were identified, 169 were descriptive and 243 interventions. Of the descriptive papers 95.0% included individual predictors, and 5.0% social predictors. None of the descriptive papers examined treatment centre variables as predictors of psychosocial outcomes. Similarly, none of the interventions evaluated the effectiveness of treatment centre interventions for improving psychosocial outcomes. Potential reasons for the overwhelming dominance of individual predictors and individual-focused interventions in psychosocial literature are discussed.

## INTRODUCTION

A diagnosis of cancer and its subsequent treatment may result in physical, emotional, psychological and spiritual distress that negatively impacts quality of life (Hewitt *et al*. 2005). This has been observed across cancer sites (Gotay & Maruoka 1998; Hewitt *et al*. 2005), patient characteristics (Carver 2005; Hewitt *et al*. 2005) and countries (Hewitt *et al*. 2005; Grunfeld 2006). Psychosocial morbidity can occur at any stage of the disease trajectory, even when the cancer has gone into remission (Gotay & Maruoka 1998; Hewitt *et al*. 2005; Grunfeld 2006).

Suggested steps for improving psychosocial outcomes for cancer patients and survivors include identifying the prevalence of psychosocial morbidity, understanding causes and predictors and intervening appropriately (Ayanian & Jacobsen 2006). Once prevalence has been established, the second step is to understand possible causes and predictors of psychosocial morbidity. There are several categories of predictors that could play a role in determining psychosocial outcomes: (1) individual variables such as demographic characteristics, traits (Ahles *et al*. 2003; Carver 2005), disease and treatment characteristics (Carlson *et al*. 2004; Bloom *et al*. 2007); (2) social variables such as social support or network of the person with cancer (Helgeson & Cohen 1996); and finally (3) treatment centre variables related to the organisation and delivery of care within a setting.

### Individual characteristics of patients and providers

Some individuals may be predisposed to experience greater psychosocial morbidity due to their psychological make-up (Ahles *et al*. 2003), demographic characteristics such as socio-economic status (Guidry *et al*. 2005) and personality traits including coping styles (Wagner *et al*. 1995). Combinations of demography and psychology may also predict psychosocial morbidity. For example, women with breast cancer who are younger and have a pre-existing history of depression have reported greater psychosocial distress than women who do not share these characteristics (Mosher & Danoff-Burg 2005). As well as individual characteristics of the patient, individual characteristics of providers may also influence outcomes for the patients they treat. Patients of providers who have undergone communication skills training may experience less distress (Fukui *et al*. 2008; Merckaert *et al*. 2008) and better coping behaviours (Fukui *et al*. 2008) than those of providers who have not undergone this type of training.

### Disease and treatment characteristics

The patient's cancer type, stage of disease and treatment (surgery, radiotherapy, chemotherapy and medication), may also predict the likelihood of experiencing poor psychosocial outcomes (Bloom *et al*. 2007). For example lung cancer patients have reported worse global quality of life scores than patients with other types of cancer (Schag *et al*. 1994). Similarly, within the same cancer diagnosis, different treatment regimes such as radiotherapy and chemotherapy versus either alone can lead to different psychosocial outcomes (Arai *et al*. 1996; Ahles *et al*. 2005).

### Social support

Social support can be defined as the provision of non-professional practical assistance, information, emotional empathy and comfort (House & Kahn 1985; Cutrona & Russell 1990) to patients by others within their social network. Research suggests that low levels of support may be linked to high levels of psychosocial morbidity (Courtens *et al*. 1996; Karnell *et al*. 2007; Knobf 2007). In a study by Parker *et al.*, cancer patients with poor social networks had worse mental functioning, higher levels of distress and lower overall quality of life, than patients with good social networks (Parker *et al*. 2003).

### Treatment centre characteristics

Features of the care environment such as procedure volume (Bach *et al*. 2001) and access to specialist care (Gillis & Hole 1996) have been associated with morbidity and mortality outcomes for patients. The association between volume and patient survival may reflect that specialists who treat many similar patients may have greater clinical skills and may be more up-to-date with best evidence (Carey *et al*. 2009). Further, because they see many similar patients they may be better systems in place to support the delivery of best evidence care for that patient group (Carey *et al*. 2009). Similarly, receipt of treatment at an institution that provides access to clinical trials has been associated with better outcomes (Newell *et al*. 1997). This has been attributed to the rigorous follow-up and care protocols that are applied to clinical trials patients (Du Bois *et al*. 2005). Given these findings, it is possible that characteristics of the treatment environment related to staff numbers, staff training, care protocol and systems may also be linked to psychosocial outcomes. Research on the role of systems may assist in developing a more sophisticated understanding of which structures and processes of care may influence psychosocial outcomes, leading to research to understand how this occurs and how such factors can be modified for the benefit of the patients served by the system.

Once prevalence and predictors have been identified, the next step is to develop and test interventions designed to ameliorate the conditions that contribute to psychosocial morbidity. While disease predictors and many demographic predictors are not modifiable, they may enable attention to be directed to those patients who are most likely to be at risk of poor psychosocial outcomes.

Some individual variables such as behaviours, coping strategies and cognitions may be modifiable. Psychological therapies aimed at modifying individual-level variables such as a patient's behaviours or cognitions, or social support have been developed (Ayanian and Jacobsen 2006; Holland & Reznik 2005). However, effect sizes are often modest (Newell *et al*. 2002; Lepore & Coyne 2006), and individual-focused interventions are often too resource intensive for routine implementation (Lepore & Coyne 2006).

There are other aspects of the cancer patient's experience that can be modified. Treatment centre variables related to the environment where care is provided are potentially modifiable (Ferlie & Shortell 2001). Through policy and practice change, characteristics of the treatment centre have potential to be modified to achieve systematic benefits for all patients who receive care within a particular setting. This suggests that the relationship between treatment centre variables and psychosocial outcomes for cancer patients is an important area of investigation.

The aim of this paper is to investigate the proportion of the published psychosocial literature over the last 10 years that has investigated the role of treatment centre variables as potential predictors of psychosocial morbidity in cancer patients. It is hypothesised that the most frequently identified predictors of psychosocial morbidity will be individual variables (individual traits, disease and treatment characteristics, etc.) followed by social support variables.

## METHODS

### Data sources and extraction

The Ovid search engine was used to search Medline for literature published between 1999 and 19 November 2009. The search string used was as follows: (cancer OR neoplasms) AND (psycho$.mp or Anxiety or Depression or Quality of Life) AND (randomised controlled trial or Intervention Studies or Cross Sectional Studies or Longitudinal Studies).

### Inclusion and exclusion criteria

Papers that reported quantitative primary data (either descriptive or intervention studies) were published in English and that were relevant to psychosocial outcomes for cancer patients were retained. Dissertations, books, case studies, qualitative studies, commentaries/letters and review papers were excluded.

Due to the large volume of publications identified, a 20% random sample was selected for classification using a random number generation. One coder categorised predictor variables for the entire sample of publications. The first author independently recoded 20% of the sample. Any disagreements were resolved by mutual discussion. Kappa statistics were calculated to determine inter-rater agreement.

### Coding of study design

Studies were initially classified as either intervention or descriptive studies. Studies were classified as descriptive if they used a cross-sectional or longitudinal design. Studies were classified as intervention if they reported on the evaluation of an intervention designed to improve psychosocial outcomes in cancer patients.

### Classification of predictor variables in descriptive studies

Publications reporting descriptive studies were examined to determine which variables were considered as potential predictors of psychosocial morbidity. Variables were grouped into four broad categories.

#### Individual predictors (patients)

This category included papers where variation in psychosocial outcomes according to individual patient characteristics was explored. Individual characteristics included the following demographic variables, traits, behaviours or experiences of the individual, disease characteristics and characteristics of the treatment received by the individual, as well as co-morbid conditions and cancer side effects.

#### Individual predictors (providers)

This category included papers where provider variables were linked to psychosocial outcomes. These could include provider demographic characteristics, attitudes, knowledge, skills or behaviours.

#### Social support predictors

This category included papers where characteristics of relationships or social networks were used to predict psychosocial outcomes, such as network size or quality of social support.

#### Treatment centre predictors

This category included papers where characteristics of the treatment centre were used to predict psychosocial outcomes. These could include both structures of care in the treatment centre where care is provided (e.g. patient volume, staff to patient ratios, equipment, services available, etc.) and processes of care used within the treatment centre (quality monitoring procedures, screening for distress, etc.). A description of variables in each of these categories is presented in Table 1.

**Table 1:** Description of predictor variable categories

Predictor category	Description
Individual predictors: characteristics of the patient	
Patients	
Demographics	Age, gender, education, marital status, socio-economic status, other demographic variables
Traits/behaviours	Coping style, self-esteem, self-efficacy, outlook, locus of control and other individual characteristics
Treatment characteristics	Treatment regimes (surgery, chemotherapy, radiation therapy, courses of specific drugs, hormone therapy); and any other variables related to the medical treatment of cancer. Including complementary therapies and psychological therapies
Disease characteristics	Cancer type, stage of cancer
Cancer side effects & co-morbid conditions	Physical and psychological consequences of the diagnosis, disease and treatment of cancer, or the presence of unrelated physical or mental health conditions, cancer-related fatigue pain, hair loss etc.
Individual-level predictors: characteristics of providers	
Providers	Demographics, attitudes, skills, knowledge of providers
Social support: characteristics of social support provided to patient	
Social support structure	Living arrangements, network size
Social support quality	Dynamics within family units, peer relationships, social networks, support from co-workers and managers, and any other variables related to social support
Treatment centre predictors: characteristics of the environment of where care is provided	
Structure of care	Volume (e.g. number of cancer patients); setting (e.g. cancer care centre); presence of a cancer training programme; presence of specific types of equipment (e.g. radiation machines); presence of and composition of a multidisciplinary team; staff to patient ratios; teaching status; and any other variables related to the structure of cancer treatment units
Process of care	Delivery of treatment; case management and decision-making; diagnosis and staging; initial clinical management; patient involvement in decision-making; referrals and coordination of care; management of treatment toxicity; use of guidelines and monitoring of best practice; surveillance after initial therapy, and any other variables related to the manner in which care is provided

#### Scoring of descriptive papers

Each paper received a score of one for each category of variable used to predict psychosocial outcomes identified within the paper. Scores for each category of predictor were summed across papers to determine the frequency of each predictor category.

### Classification of interventions

Intervention studies were coded according to whether they sought to modify characteristics of the individual patient or provider, characteristics of the patient's relationships or social support, or characteristics of the treatment centre.

#### Individual patient-focused

Interventions aimed at changing the knowledge, attitudes, traits, cognitions, behaviours or treatment of the cancer patient. In these studies, the unit of intervention and analysis was the patient. Studies that examined medical treatments that included psychosocial outcomes as either a primary or secondary outcome were included. These studies are denoted as individual patient-focussed (medical).

#### Individual provider-focused

Interventions aimed at changing provider knowledge, attitudes or behaviour including communication skills without changing any other aspect of the care environment. In these studies the unit of intervention was the provider, however, outcomes for individual patients were measured. Interventions aimed at assisting the provider with assessment of needs were included here.

#### Social support-focused

Intervention aimed at changing relationships or support structure within a family or other small social network. Interventions aimed at improving doctor–patient communication were included in this category if the intervention targeted the doctor's and the patient's behaviour, knowledge or skills. In these studies the unit of intervention and analysis was the social unit (e.g. family, dyad, etc.).

#### Treatment centre-focused

Interventions aimed at changing the structure, organisation or delivery mechanism of care. In these studies the unit of intervention and analysis was the system of care (e.g. hospital, clinic or ward).

#### Scoring of intervention papers

A score of 1 was assigned for each foci of the intervention (individual patient, individual provider, social environment, treatment centre). Scores in each category of intervention were summed across papers to give the frequency of each predictor category.

### Statistical analysis

PASW Statistics 18.0 was used to test the distribution of predictor variables across both the intervention and descriptive papers. *χ*^2^-tests were used to test the hypothesis that patient-related characteristics would be the most frequently reported predictors of psychosocial outcomes.

## RESULTS

A total of 4453 publications were identified in the literature search. From these, a 20% sample of publications was randomly selected. Of these 891 papers, 479 (53.8%) papers did not meet the inclusion criteria. Of the excluded papers, 214 (44.7%) were irrelevant to psychosocial outcomes, 134 (28.0%) were not focused on cancer patients, 71 (14.8%) did not report quantitative primary data, 43 (9.0%) were not published in English, and 17 (3.5%) were duplicates. The remaining 412 publications were coded for both study type and predictor type. For classification of included papers, inter-rater agreement as determined by kappa statistic was 0.82.

Of the 412 papers that were included for further coding, 169 (41.0%) were classified as descriptive and 243 (59.0%) as interventions. The descriptive studies were further coded to indicate the type of predictor variables used to predict psychosocial outcomes. The number of descriptive papers reporting each type of predictor is shown in Table 2. A significantly greater number of papers examined individual predictors compared with those which examined social or treatment centre predictors (*χ*^2^= 212.0, d.f. = 3, *P*= 0.005).

**Table 2:** Number of descriptive studies reporting each descriptor category

Predictor type	Number (%)
Individual	
Demographics	56 (16.7)
Traits/behaviours/experiences	56 (16.7)
Disease	48 (14.3)
Treatment	77 (22.9)
Co-morbid conditions and cancer side effects	82 (24.4)
Individual level (provider)	0 (0.0)
Social support	
Support structure	1 (0.3)
Support quality	16 (4.7)
Treatment centre characteristics	
Structure of care	0 (0.0)
Process of care	0 (0.0)

The distribution of intervention types can be found in Table 3. As with the descriptive studies, the number of studies evaluating interventions to modify individual characteristics was significantly greater than the number evaluating interventions to change social or treatment centre characteristics (*χ*^2^= 145.67, d.f. = 4, *P*= 0.005). Of the 241 interventions concentrating on individual-level variables, 97 (40.2%) used psychosocial strategies, 140 (58.1%) used medical techniques, and 4 targeted the provider (1.7%).

**Table 3:** Number of interventions by primary focus of intervention

Intervention type	Number (%)
Individual-focused (patient) (provider)	241 (99%)
Social support-focused	2 (0.8)
Treatment centre-focused	0 (0.0)

## DISCUSSION

This review sought to identify the proportion of psychosocial literature that has examined the potential role of treatment centre variables as predictors of psychosocial morbidity in cancer patients. No descriptive or intervention studies relevant to treatment centre predictors of psychosocial well-being were identified among the studies reviewed. This is surprising, given that strong attention has been directed towards the importance of health services as a predictor of other health outcomes including survival (Gillis & Hole 1996; Bach *et al*. 2001; Du Bois *et al*. 2005).

### What accounts for the predominant focus on individual-level predictors?

Given that the majority of research focuses on individual predictors of psychosocial well-being, it is important to consider what factors might underlie this. These may include: (1) the theoretical orientation and training of the psychosocial researcher; (2) the greater availability of measures of individual variables compared with system variables; (3) the attention to individual-focused interventions in psychology practice; and (4) the practical advantages of doing individual-focused research compared with system-focused research.

#### Theoretical orientation of psychosocial researchers leads to a focus on individual and social support predictors rather than a treatment centre focus

Given that many psychosocial researchers have a psychology background (Nehl *et al*. 2003), it is plausible that the individual focus in psychological theories and training has contributed to the preponderance of psycho-oncology research focused on individual predictors of distress. While psychological theories acknowledge the role of a range of factors in the aetiology of psychological distress, the emphasis is on the role of factors such as cognitions, attitudes, behaviours (Beck *et al*. 1979), interpersonal relationships (Ravitz *et al*. 2008) and social factors (Panzarella *et al*. 2006). This orientation toward individual and, to a lesser extent, social predictors of human behaviour is reflected in the training of psychology professionals (Ewart 1991).

#### Measures of individual characteristics are more readily available than measures of treatment centre characteristics

The interest in individual predictors has lead to the development of a number of standardised measures that can be used to measure individual variables (Spielberger *et al*. 1983; Folkman & Lazarus 1988; Endler & Parker 1990; Sherbourne & Stewart 1991; Weinman *et al*. 1996). Such tools are widely used and accepted by researchers. In contrast there is a dearth of established methods and measures to assess treatment centre characteristics (Moos *et al*. 1973). Further, there is likely to be much less agreement about what types of treatment centre characteristics may be important to assess. Where measures do exist, for example, ward climate scales (Moos *et al*. 1973) these are likely to be less well known and accepted than measures of individual variables. Therefore, the greater availability of well established and validated tools to assess individual predictors in comparison with treatment centre predictors may perpetuate the focus on individual factors.

#### Individual measures lead to individual-focused rather treatment centre-focused interventions

If psychosocial research focuses only on the measurement of individual predictors, then these are the only explanatory factors available for the researcher to interpret his or her findings. This will lead naturally to the development of interventions aimed at modifying individual characteristics such as cognitions and behaviours. Congruence between theory and intervention is widely advocated (Bonetti *et al*. 2006; Michie *et al*. 2008), therefore factors that fall outside the theoretical orientation and expertise of those who do the research, such as systems of care, are not likely to be considered as avenues for intervention. This leads to evaluation of individual-focused interventions, thereby contributing to the focus on the individual rather than the system of care.

#### Individual-focused research is easier to do than treatment centre-focused research

As cancer is a common disease [Australian Institute of Health and Welfare *et al*. 2008; World Health Organization (WHO) 2004], there are likely to be few problems associated with accessing an appropriate sample of patients for research. As discussed, the assessment of individual variables in such research is commonly accepted and widely practiced. In contrast to this, a focus on system factors would lead to considerable challenges for researchers. These may relate to logistical and cost considerations related to obtaining a large enough sample of treatment centres with which to assess the role of system factors (Mercer *et al*. 2007; Sanson-Fisher *et al*. 2007). There may also be significant political sensitivities associated with the collection of data related to treatment centre characteristics, especially where there may be implications for professional and institutional reputations. These factors suggest that it is easier to do individual-focused research than research focused on the system of care. This may contribute to the continued research focus on individual predictors.

### Why should characteristics of the treatment centre be examined as possible predictors of psychosocial outcomes?

Treatment centre factors relate to the characteristics of the organisation where care is provided (Donabedian 2005) and may also relate to the broader health care context. Treatment centre characteristics may create an environment that influences the practice of providers (Institute of Medicine 2001). This influence may be exerted through policies, procedures and performance monitoring (Carey *et al*. 2009). In this way the system defines what is expected from providers (Donabedian 1997). As such treatment centre factors can help improve patient outcomes by supporting practices and processes of care that are linked to better outcomes (Donabedian 1997). If we accept that these principles operate to influence outcomes in psychosocial care, then this suggests a need to examine the role of structures and processes of care as predictors of psychosocial outcomes.

Treatment centre variables have the potential to provide fruitful avenues for investigation and intervention in improving a range of outcomes (Von Korff *et al*. 1997), including psychosocial morbidity. There are several advantages to exploring the role of treatment centre variables in psychosocial outcomes. First, many individual demographic or disease variables such as age, gender, type of cancer and stage are non-modifiable. Personality traits such as extraversion or optimism are also difficult to modify. Individual psychological characteristics such as coping strategies and behaviours may be modified; however, uptake of interventions may be low and effects modest (Lepore & Coyne 2006). To optimise uptake and effectiveness of individual-focused psychological interventions, implementation will need to be supported by systems within the treatment centre (e.g. training, policies, procedures, coordination of care). Similarly, intervention aimed at changing the practice of individual providers needs to be coupled with strategies at the organisational or treatment centre level (Grol 2002). This is because organisational factors may support or hinder systematic implementation of best practice (Grol 2002). Systems to monitor patient outcomes or relevant processes of care may be costly to develop and maintain (Donabedian 1997). Hence an approach that enables a range of potential problems to be assessed may be more efficient that multiple individual-focused interventions or services developed and implemented in isolation. Systematic approaches for assessing anxiety, depression as well as a range of other concerns related to information, physical, spiritual and emotional well-being have been trialled previously (McLachlan *et al*. 2001).

A focus on individual predictors suggests that the burden of change to improve psychosocial outcomes rests with the person with cancer. This is at odds with the paradigm employed in other areas of medicine; whereby variation in outcomes are seen as resulting not only from variation in clinical variables, but also from the quality of care received and the treatment centre characteristics that support delivery of quality care (Grol *et al*. 2002). The latter approach places the onus on the treatment delivery setting rather than the individual patient to ensure that the best possible outcomes are achieved for the individual. Third, an approach that takes into account the role of treatment centre characteristics is likely to support equitable care delivery. Adoption of the approach increases the likelihood that all patients, regardless of which provider they see and how well they can communicate their needs, will have access to the best practice psychosocial care.

### How can characteristics of the treatment centre be measured?

There are a number of existing approaches available for considering the effect of treatment centres. Donabedian's model involves the considering the role of ‘structures’ and ‘processes of care’ on patient outcomes (Donabedian 2005). Structural variables refer to characteristics of the organisation that facilitate delivery of high quality care. These may include size of the organisation, equipment or number of staff (Brien *et al*. 2009). The process domain covers variables related to delivery of care including the presence of policies, procedures and cues to support implementation of best practice care (Brien *et al*. 2009).

Other approaches have emphasised the importance of factors such as organisational culture and climate (Moos *et al*. 1973; Bosch *et al*. 2008), team functioning (Ouwens *et al*. 2008) and leadership (Rhydderch *et al*. 2004). Culture and climate relate to a team's shared values and beliefs about an organisation's policies and practices (Hann *et al*. 2007). These factors are thought to create conditions conducive to the adoption of best practice care (Greenhalgh *et al*. 2004).

The National Health Service in the UK assesses consumer perceptions of the following domains of care: (1) responsiveness to consumer needs, values and preferences; (2) integration and coordination; (3) physical comfort; (4) emotional support; (5) involvement of family and friends; and (6) information, communication and education (Jenkinson *et al*. 2002). These criteria were developed from consumer views about what is important to quality of care (Gerteis *et al*. 1993). Notably this focuses predominantly on process of care rather than on structures or team functioning aspects. This perhaps reflects that the latter factors are less likely to be observable to consumers.

Each of the approaches to examining treatment centre characteristics leads to different types of data collection strategies. Donabedian's model focuses on variables that can be assessed from administrative data. Some process of care data may be examined via medical records audit or through patient or provider report (Donabedian 2005). Assessment of organisational cultural may be done by key informant interviews (Donabedian 2000; Donabedian 2005), or surveys of staff within the institution (Bosch *et al*. 2008). In contrast, the NHS approach puts an emphasis on the views of consumers (Jenkinson *et al*. 2002; Healthcare Commission 2005). While each of these theoretical approaches discussed have limitations, each represents a starting point for beginning to examine the role of system characteristics in psychosocial outcomes.

### How can variation in outcomes between treatment centres be assessed?

The first step in assessing whether variation in outcomes is related to treatment centre characteristics is to determine the level of variation in psychosocial outcomes between organisations. This requires the use of a reliable and valid measure of psychosocial well-being (Kirshner & Guyatt 1985). The measure needs to be administered to a randomly selected number of patients in each treatment centre. The number of patients should be sufficient to represent the performance of the treatment centre. If variation exists, then the relative contribution of patient and treatment centre variables can be examined.

### How can interventions to modify treatment centre characteristics occur?

If system factors are shown to influence psychosocial outcomes, then a strategy for intervening is needed. Interventions for changing systems of care include the use of local opinion leaders to influence the culture and practices of others within the organisation (Doumit *et al*. 2006); the use of audit and feedback (Jamtvedt *et al*. 2006); and implementation of policies and procedures (Rubenstein *et al*. 2000). A commonly used approach is the collaborative method (Wagner *et al*. 2001). This involves collection of outcome or process of care data and regular provision of feedback to the clinical team. The team is responsible for setting performance improvement goals and identifying where care can be improved (Wagner *et al*. 2001). One of the appealing characteristics of this intervention strategy is that it allows some flexibility for intervention strategies to be tailored to the needs of each participating organisation. More research is needed, however, to develop evidence of effectiveness (Schouten *et al*. 2008).

### How can interventions to modify treatment centre characteristics be evaluated?

Where the unit of intervention is the system not the individual patient, randomised controlled trials in which individual patients are allocated to the intervention or control group may be unsuitable (Mercer *et al*. 2007). Cluster randomised controlled trials where the unit of allocation is the organisation may be used as an alternative (Mercer *et al*. 2007; Sanson-Fisher *et al*. 2007). Data are collected from a sufficient number of patients within each organisation to enable the performance of the organisation to be represented. An adequate sample of both organisations and patients therefore needs to be recruited (Sanson-Fisher *et al*. 2007).

Often, however, it is not feasible to recruit the number of organisations needed for a cluster randomised trial (Mercer *et al*. 2007; Sanson-Fisher *et al*. 2007). The Cochrane Effective Practice and Organization of Care Group recommends in addition to randomised trial, controlled before and after studies and interrupted time series designs are appropriate for this type of evaluation (Bero *et al*. 2002). Controlled before and after studies involve a control group and intervention group that are not randomly assigned (Bero *et al*. 1997). Baseline and post-test data collection must be collected at the same time in both groups.

Interrupted time series studies involve collection of repeated measures data in one site. At least three data points must be collected both before and after the intervention to establish whether there is any change of trend in the data due to the intervention (Bero *et al*. 1997). A variation to this is the multiple baseline design (Hawkins *et al*. 2007). This involves collection of repeated measures data in several sites. The timing of the intervention is staggered between sites to allow greater control for the effect of external variables on any changes in trend observed (Hawkins *et al*. 2007).

Understanding the prevalence and predictors of psychosocial morbidity, and intervening appropriately, are critical requirements to improve cancer outcomes. This study examined a 20% random sample of psychosocial research literature published over the last 10 years to determine the extent to which research has examined individual-level, social and treatment system predictors. The majority of both descriptive and intervention studies focused on individual-level variables; only 5.0% of descriptive and 0.8% of intervention studies addressed social support variables, and none examined treatment centre predictors. Possible reasons for this discrepancy and suggestions for future research were proposed.

### Study limitations

The literature search was conducted using one electronic database and only peer-reviewed papers were included. The search terms used in the review are commonly employed in the psycho-oncology literature; however, it is possible that some relevant articles were missed by these terms. While it is possible that a broader search using additional databases and inclusion of grey literature would have identified additional articles relevant to this review, it is unlikely that this would have substantially changed the proportion of papers examining each type of predictor.

### Conclusions

Few studies have examined the role of treatment centre characteristics in psychosocial outcomes for cancer patients. There is a need to rigorously assess what types of treatment centre characteristics may influence outcomes and to what degree. This creates a potential avenue for developing interventions aimed improving patient outcomes although the implementation of cohesive and systematic processes and structures to support best practice psychosocial care.
